# Development of an HDL-C combined with AJCC staging system for predicting overall survival after curative resection of intrahepatic cholangiocarcinoma without metabolic comorbidities

**DOI:** 10.3389/fonc.2026.1884360

**Published:** 2026-08-07

**Authors:** Chengzhi Jiang, Kaijun Long, Qian Ma, Fang Cheng, Ji Tao, Kaiguo Long

**Affiliations:** 1 Department of Breast and Thyroid Surgery, The Second Affiliated Hospital of Zunyi Medical University, Zunyi, Guizhou, China; 2 Department of Gastrointestinal Medical Oncology, Harbin Medical University Cancer Hospital, Harbin, Heilongjiang, China; 3 Department of Critical Care Medicine, Wenjiang District People’s Hospital, Chengdu, China; 4 Department of General Surgery, The Second People’s Hospital of Yibin, Yibin, Sichuan, China

**Keywords:** AJCC staging system, HDL-C, intrahepatic cholangiocarcinoma, lipid metabolism, nomogram

## Abstract

**Purpose:**

To develop and validate a prognostic model integrating HDL-C and AJCC staging system for predicting overall survival (OS) in patients with intrahepatic cholangiocarcinoma (ICC) following curative resection without metabolic comorbidities.

**Patients and methods:**

A total of 291 with ICC who underwent curative resection between June 2013 and November 2024 were retrospectively enrolled and randomly assigned to a training cohort (n=203) and a validation cohort (n=88). Receiver operating characteristic (ROC) curve analysis was performed to identify the optimal serum lipid biomarkers. Independent prognostic factors for OS were identified using univariate and multivariate Cox proportional hazards regression analyses. A nomogram incorporating clinicopathological characteristics and serum lipid parameters was subsequently developed and internally validated using calibration curves, time-dependent ROC curves, the concordance index (C-index), the Hosmer–Lemeshow goodness-of-fit test, and decision curve analysis (DCA).

**Results:**

LDL-C and HDL-C demonstrated favorable predictive performance, with AUC values of 0.9012 and 0.8557, respectively. Multivariate Cox regression analysis identified low body mass index, low HDL-C, smoking, lymphatic invasion, perineural invasion, and AJCC stage as independent adverse prognostic factors for OS. The nomogram demonstrated good predictive performance, with 1-, 2-, and 3-year AUC values of 0.866, 0.801, and 0.721, respectively, and a C-index of 0.701 in the training cohort. Calibration curves showed good agreement between predicted and observed survival probabilities, while DCA confirmed favorable clinical utility. Internal validation further showed good discriminative ability, with an AUC of 0.811.

**Conclusion:**

A modified prognostic model integrating HDL-C with the AJCC staging system was successfully developed and validated for predicting overall survival in patients with ICC who underwent curative resection. Low HDL-C was identified as an independent adverse prognostic factor, suggesting that altered lipid metabolism may contribute to ICC progression. This model may provide a practical tool for individualized prognostic assessment and clinical decision-making.

## Introduction

Over the past decades, the incidence of ICC has increased steadily worldwide, with 5-year overall survival rate ranging from 7% to 20% (1). Surgical resection is currently the only potentially curative treatment for ICC. However, due to the high heterogeneity of this malignancy, only 20%–30% of patients are eligible for surgery. Moreover, even after radical resection, long-term survival varies considerably and is influenced by multiple clinicopathological factors, including tumor differentiation, tumor size, and lymphovascular invasion (2).

Although the American Joint Committee on Cancer (AJCC) staging system remains the most widely used tool for prognostic assessment and clinical decision-making in ICC, it is based primarily on anatomical tumor characteristics and therefore does not fully reflect the biological behavior or tumor microenvironment heterogeneity of ICC (3). Therefore, identifying reliable prognostic biomarkers to complement the AJCC staging system may improve the prediction of overall survival and facilitate individualized clinical decision-making for ICC patients underwent surgery.

Recent studies have identified several promising prognostic biomarkers for ICC, including the prognostic inflammatory and immunonutritional index (PIIN), tumor-associated tertiary lymphoid structures (TLSs), and the high endothelial venule (HEV) subtype (4–6). However, their clinical application remains limited because of relatively complex assessment procedures and the lack of standardized evaluation criteria. In contrast, serum lipids are routinely measured biochemical parameters in clinical practice and play essential roles in energy metabolism and intracellular signaling. Increasing evidence from metabolomic studies has demonstrated that dysregulated lipid metabolism is closely associated with tumor initiation, progression, and prognosis (7). Cancer cells undergo extensive metabolic reprogramming, characterized by enhanced *de novo* lipid synthesis and altered fatty acid metabolism to support rapid proliferation and surviva (8–10). These findings suggest that serum lipid profiles may serve as practical and biologically relevant biomarkers for cancer prognosis.

Increasing evidence suggests that serum lipid profiles are associated with cancer prognosis. A retrospective study showed that low triglyceride and low HDL-C levels were significantly associated with thyroid cancer recurrence (11). In addition, serum triglyceride and HDL-C levels were correlated with disease severity in prostate cancer (12). Furthermore, large-scale lipidomics study further identified a causal association between elevated LDL-C levels and gastric cancer risk, while triglyceride levels ≥2.2 mmol/L were associated with an increased risk of gallbladder cancer in men aged over 60 years (13). However, the prognostic value of serum lipid profiles in patients with ICC undergoing curative resection remains largely unexplored (14, 15).

**Figure 1:**
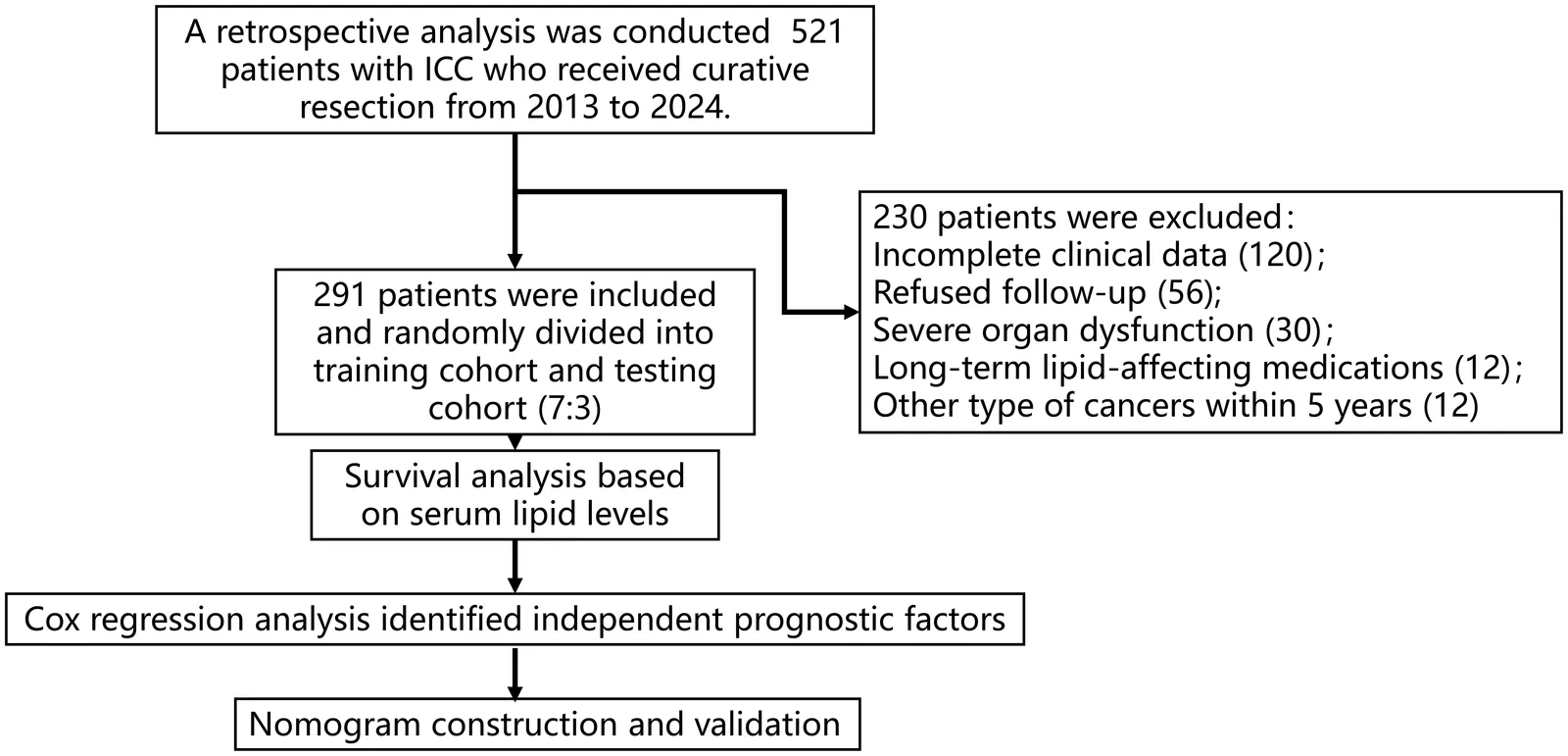
Flow chart of the study.

The present study aimed to develop and validate a prognostic model integrating serum lipid parameters with the AJCC staging system to improve the prediction of postoperative overall survival in patients with ICC, thereby facilitating individualized prognostic assessment and clinical decision-making (**Figure 1**).

## Methods

### Study population

Patients diagnosed with ICC who were treated at our center between June 2013 and November 2024 were retrospectively enrolled in this study. All eligible patients were randomly assigned to a training cohort and a validation cohort at a ratio of 7:3 by simple randomization. The inclusion criteria were as follows: (1) pathologically confirmed ICC; (2) receipt of curative surgical resection; (3) availability of serum lipid measurements within 1 week before surgery; (4) at least one valid postoperative efficacy evaluation; (5) an Eastern Cooperative Oncology Group (ECOG) performance status score of 0–2; (6) no receipt of neoadjuvant or adjuvant therapy; and (7) age between 18 and 80 years.

The exclusion criteria were as follows: (1) incomplete clinical data or refusal to undergo follow-up; (2) absence of pathological confirmation; (3) diagnosis of other malignancies before surgery or within 5 years after surgery; (4) receipt of non-curative surgery or local interventional therapy; (5) presence of severe organ dysfunction; (6) coexistence of chronic diseases that could significantly affect serum lipid levels, such as diabetes mellitus or steatohepatitis; and (7) use of medications affecting serum lipid metabolism within 1 month before surgery, including statins and fibrates.

### Clinical data collection

Clinical records were retrospectively reviewed to collect demographic, clinicopathological, and follow-up data, including sex, age, height, weight, date of diagnosis, date of surgery, follow-up duration, surgical and pathological findings, and established high-risk pathological features, including vascular invasion, lymphatic invasion, serosal invasion, multiple tumors, satellite nodules, lymph node metastasis, and histological differentiation. In addition, preoperative total bilirubin levels within 1 week before surgery, long-term smoking history, alcohol abuse history, ECOG performance status, and AJCC stage were also recorded.

Furthermore, preoperative serum lipid profiles obtained within 1 week before surgery were collected for all patients, including low-density lipoprotein cholesterol (LDL-C), high-density lipoprotein cholesterol (HDL-C), total cholesterol (CHOL), triglycerides (TG), apolipoprotein B (ApoB), apolipoprotein A-1 (ApoA-1), and lipoprotein(α) (Lp(α)).

### Missing data

The data was deleted which the absence exceeded 50% in all patients. If the absence under 50%, we filled it with 1/5 of the minimum value among the factor.

### Baseline analysis

To ensure comparability between groups and improve the accuracy of prognostic evaluation, baseline analyses were performed before formal statistical analyses. All collected patient data were analyzed using the “TableOne” package, and corresponding p values were calculated. P value <0.05 was considered statistically significant.

### Screening of serum lipid biomarkers

To identify the optimal serum lipid biomarkers for prognostic model construction, receiver operating characteristic (ROC) curves were generated using the “pROC” package. The area under the curve (AUC), hazard ratio (HR), 95% confidence interval (95% CI), optimal cutoff value, sensitivity, and specificity were subsequently calculated. Biomarkers with an AUC >0.7 were selected for subsequent survival analyses, Cox regression analyses, and prognostic model development.

### Survival analysis and follow-up

Computed tomography (CT) was used to evaluate preoperative tumor size and status, as well as for postoperative follow-up assessments. OS was defined as the interval from the date of diagnosis to death from any cause or the date of the last follow-up, which was calculated according to the date of diagnosis, date of surgery, and follow-up time. Subsequently, survival analyses were performed using the “survival” package to calculate median OS (mOS), HRs, 95% CIs, and p values. P value <0.05 was considered statistically significant. Kaplan–Meier survival curves were then generated using the “ggplot2” package.

### Univariate and multivariate Cox regression analysis

To investigate the associations between clinicopathological characteristics, serum lipid biomarkers, and OS, univariate and multivariate Cox regression analyses were performed using the “survival” package. Variables identified in the multivariate analysis were considered independent prognostic factors for OS. Forest plots were subsequently generated using the “forestplot” package.

### Nomogram construction and validation

Independent prognostic factors identified from multivariate Cox regression analyses were incorporated into the nomogram construction. The nomogram was established using the “rms” package. Calibration curves were subsequently generated to evaluate the calibration performance of the model, while the “timeROC” package was used to assess the discriminatory ability of the model. The concordance index (C-index) was calculated to evaluate model consistency, and the Hosmer–Lemeshow test was performed to assess model goodness-of-fit. In addition, decision curve analysis (DCA) was conducted using the “Dcurves” package to evaluate the clinical net benefit of the model.

Furthermore, internal validation was performed using data from the validation cohort to assess the predictive performance of the model in an independent dataset. Calibration curves and ROC analyses were similarly applied during the internal validation process.

### Statistical analysis

All statistical analyses were performed using R software (version 4.3.2) for data processing, statistical analysis, table generation, and figure visualization. Categorical variables were expressed as frequencies (percentages). Normally distributed continuous variables were presented as mean ± standard deviation (SD), whereas non-normally distributed continuous variables were expressed as median (interquartile range, IQR). Fisher’s exact test was used for comparisons of categorical variables between groups.

## Results

### Clinical characteristics of participants

A total of 521 consecutive patients with ICC who underwent curative resection were initially screened for eligibility. After applying the predefined inclusion and exclusion criteria, 291 patients were included in the final analysis, whereas 230 patients were excluded, yielding an exclusion rate of 44%. The eligible patients were randomly assigned to a training cohort (n = 203) and a validation cohort (n = 88) at a ratio of 7:3. The baseline clinicopathological characteristics were well balanced between the training and validation cohorts, except for ECOG performance status (p = 0.025) (**Table 1**). The sensitivity analysis was used to evaluate this imbalance by multivariate Cox analysis adding ECOG, which demonstrated that C-index was 0.692 and all factors did not significant difference compared to those who did not join ECOG (**Supplementary Table 1**). The median follow-up duration for the entire cohort was 2.43 years.

**Table 1:** Clinical characteristics of ICC.

Characteristics	Overall	Training cohort	Validation cohort	*p*-value
	291	203	88	
BMI [mean (SD)]	22.24 (3.73)	22.23 (3.70)	22.26 (3.81)	0.957
BIL μmol/L [median (IQR)]	15.88 [11.38, 26.77]	15.50 [11.22, 26.90]	16.59 [11.90, 26.40]	0.699
Age [mean (SD)]	57.47 (8.72)	57.83 (8.36)	56.65 (9.48)	0.29
Tumor size (cm) [median (IQR)]	6.00 [4.00, 8.00]	6.00 [4.00, 8.00]	6.00 [3.00, 8.00]	0.524
Gender = Male (%)	178 (61.2)	122 (60.1)	56 (63.6)	0.602
Smoking = Yes (%)	48 (16.5)	39 (19.2)	9 (10.2)	0.061
Drinking = Yes (%)	39 (13.4)	32 (15.8)	7 (8.0)	0.091
ECOG (%)				**0.025**
0	46 (15.8)	30 (14.8)	16 (18.2)	
1	242 (83.2)	173 (85.2)	69 (78.4)	
2	3 (1.0)	0 (0.0)	3 (3.4)	
Histological grade (%)				0.851
Well	32 (11.0)	24 (11.8)	8 (9.1)	
Well-moderate	25 (8.6)	17 (8.4)	8 (9.1)	
Moderate	123 (42.3)	85 (41.9)	38 (43.2)	
Moderate-poor	48 (16.5)	31 (15.3)	17 (19.3)	
Poor	63 (21.6)	46 (22.7)	17 (19.3	
Child-Pugh grade = B (%)	161 (55.3)	105 (51.7)	56 (63.6)	0.072
CEA ≤ 4 µg/L (%)	173 (59.7)	121 (59.9)	52 (59.1)	0.897
CA199 ≤ 20 U/mL (%)	162 (55.7)	118 (58.1)	44 (50.0)	0.248
Vascular Invasion = Yes (%)	125 (43.3)	86 (42.6)	39 (44.8)	0.796
Lymphatic vessel Invasion = Yes (%)	75 (25.8)	57 (28.1)	18 (20.5)	0.191
Nerve Invasion = Yes (%)	115 (39.5)	84 (41.4)	31 (35.2)	0.362
Serous membrane Invasion = Yes (%)	149 (51.2)	100 (49.3)	49 (55.7)	0.372
Multiple Tumors = Yes (%)	72 (24.7)	51 (25.1)	21 (23.9)	0.883
Satellite Nodules = Yes (%)	90 (31.0)	65 (32.0)	25 (28.7)	0.678
Lymphatic Metastasis = Yes (%)	100 (34.4)	68 (33.5)	32 (36.4)	0.687
AJCC (%)				0.433
I	94 (32.3)	61 (30.0)	33 (37.5)	
II	111 (38.1)	79 (38.9)	32 (36.4)	
III	86 (29.6)	63 (31.0)	23 (26.1)	

*p-value<0.05* is statistically significant. BMI, Body Mass Index; BIL, Bilirubin; ECOG, Eastern Cooperative Oncology Group; CEA, Carcinoma Embryonic Antigen; CA199, Carbohydrate Antigen 199; AJCC, American Joint Committee on Cancer.

The bold values showed that the values have statistical significance.

### Screening of serum lipid biomarkers

**Figure 2:**
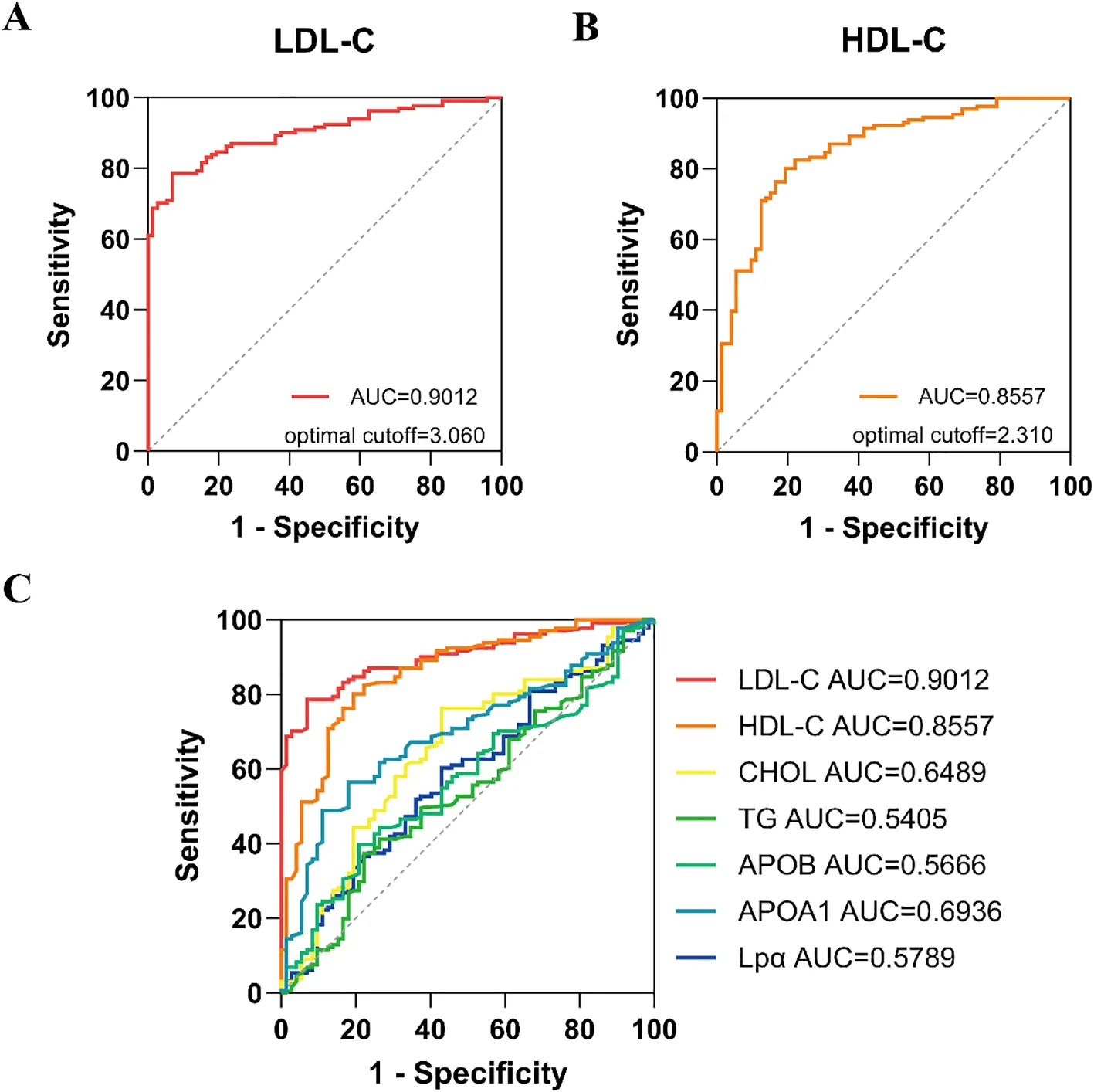
The ROC of serum lipid levels for training cohort. **(A)** The ROC of LDL-C levels. **(B)** The ROC of HDL-C levels. **(C)** The ROC of total serum lipid levels. ROC, Receiver operating characteristic. LDL-C, Low-Density Lipoprotein Cholesterol. HDL-C, High-Density Lipoprotein Cholesterol.

Subsequently, ROC curve analyses were performed in the training cohort to identify serum lipid biomarkers, and lipid parameters with an AUC > 0.7 were selected for subsequent prognostic model construction. The ROC analysis identified LDL-C and HDL-C as the only serum lipid parameters with AUC values exceeding 0.7. LDL-C showed the highest predictive performance, with an AUC of 0.9012 (95% CI: 0.860–0.942) (**Figure 2A**), whereas HDL-C achieved an AUC of 0.8557 (95% CI: 0.802–0.910) (**Figure 2B**). The remaining serum lipid parameters, all with AUC values below 0.7, were excluded from subsequent analyses(**Figure 2C**).

### Survival analysis according to LDL-C and HDL-C levels

**Figure 3:**
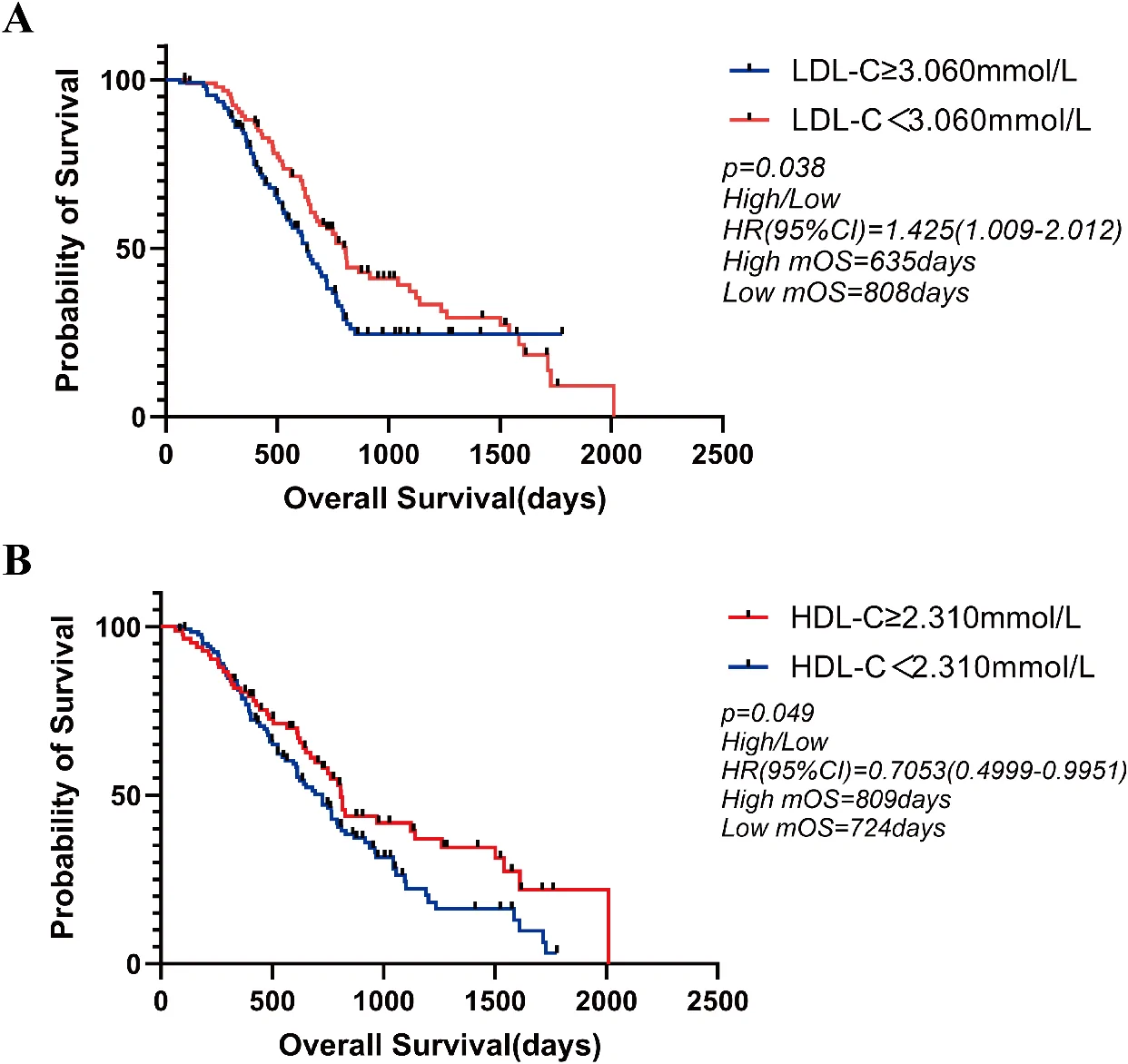
The Kaplan-Meier curves of OS for training cohort. **(A)** The Kaplan-Meier curve of OS for LDL-C. **(B)** The Kaplan-Meier curve of OS for HDL-C. LDL-C, Low-Density Lipoprotein Cholesterol. HDL-C, High-Density Lipoprotein Cholesterol. OS, overall survival.

We next evaluated overall survival (OS) according to LDL-C and HDL-C levels. Kaplan–Meier survival analysis revealed significant differences in OS between both LDL-C and HDL-C subgroups. Patients with low LDL-C levels had a median OS that was 5.8 months longer than those with high LDL-C levels (**Figure 3A**), whereas patients with high HDL-C levels had a median OS that was 2.8 months longer than those with low HDL-C levels (**Figure 3B**). These results suggest that lower LDL-C and higher HDL-C levels were associated with improved survival.

### Cox regression analysis

**Figure 4:**
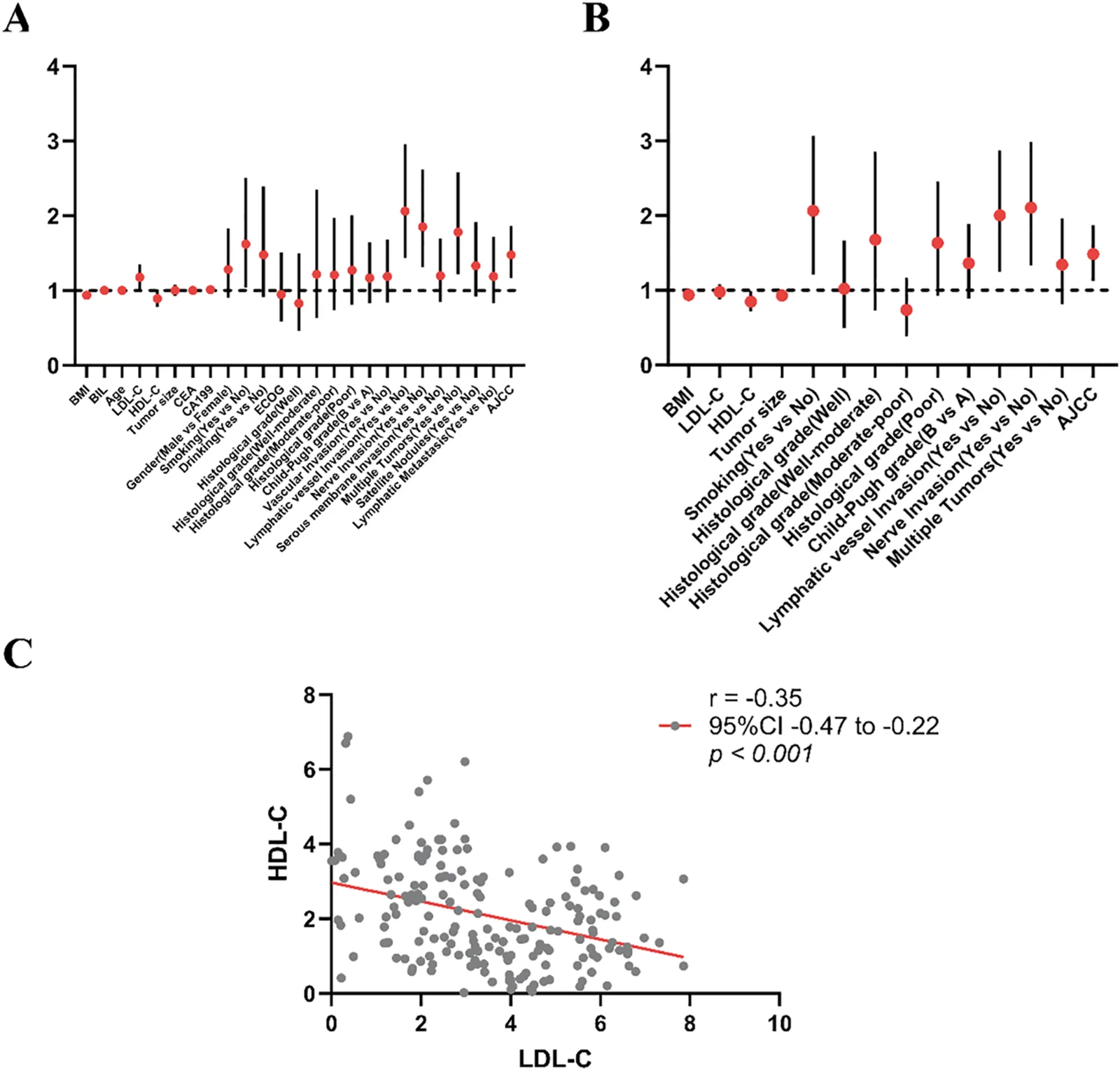
The COX regression analysis of training cohort. **(A)** The univariate analysis of OS. **(B)** The multivariate analysis of OS. **(C)** The correlation between LDL-C and HDL-C. Notes: LDL-C, Low-Density Lipoprotein Cholesterol; HDL-C, High-Density Lipoprotein Cholesterol; OS, overall survival; p<0.05 showed statistically significant.

Subsequently, LDL-C, HDL-C, and other clinicopathological variables were included in Cox regression analyses. Univariate Cox regression identified BMI and HDL-C as favorable prognostic factors, whereas LDL-C, smoking, lymphatic invasion, perineural invasion, multiple tumors, and AJCC stage were adverse prognostic factors for OS (**Figure 4A**; **Table 2**). Multivariate Cox regression further demonstrated that low BMI, low HDL-C, smoking, lymphatic invasion, perineural invasion, and AJCC stage were independent prognostic factors for OS (**Figure 4B**; **Table 3**).

**Table 2:** Univariate COX regression analysis of OS.

Characteristics	*p*-value	HR (95%CI)
BMI	**0.009**	0.94 (0.9-0.99)
BIL	0.071	1 (1-1)
Age	0.903	1 (0.98-1.02)
LDL-C	**0.048**	1.12 (0.89-1.35)
HDL-C	**0.042**	0.89 (0.78-1.01)
Tumor size	0.995	1 (0.93-1.08)
CEA	0.545	1 (0.98-1.01)
CA199	0.701	1.01 (0.97-1.04)
Gender (Male vs Female)	0.174	1.28 (0.9-1.83)
Smoking (Yes vs No)	**0.033**	1.62 (1.04-2.51)
Drinking (Yes vs No)	0.112	1.48 (0.91-2.39)
ECOG	0.824	0.95 (0.59-1.51)
Histological grade (Well)	0.529	0.83 (0.46-1.5)
Histological grade (Well-moderate)	0.554	1.22 (0.63-2.35)
Histological grade (Moderate-poor)	0.443	1.21 (0.74-1.97)
Histological grade (Poor)	0.294	1.27 (0.81-2.01)
Child-Pugh grade (B vs A)	0.374	1.17 (0.83-1.65)
Vascular Invasion (Yes vs No)	0.326	1.19 (0.84-1.68)
Lymphatic vessel Invasion (Yes vs No)	**<0.001**	2.06 (1.44-2.96)
Nerve Invasion (Yes vs No)	**<0.001**	1.85 (1.31-2.62)
Serous membrane Invasion (Yes vs No)	0.297	1.2 (0.85-1.7)
Multiple Tumors (Yes vs No)	**0.002**	1.78 (1.22-2.58)
Satellite Nodules (Yes vs No)	0.124	1.33 (0.92-1.92)
Lymphatic Metastasis (Yes vs No)	0.348	1.19 (0.83-1.72)
AJCC	**0.001**	1.48 (1.17-1.86)

BMI, Body Mass Index; BIL, Bilirubin; ECOG, Eastern Cooperative Oncology Group; CEA, Carcinoma Embryonic Antigen; CA199, Carbohydrate Antigen 199; AJCC, American Joint Committee on Cancer; LDL-C, Low-Density Lipoprotein Cholesterol; HDL-C, High-Density Lipoprotein Cholesterol; OS, overall survival. *p-value<0.05* is statistically significant.

The bold values showed that the values have statistical significance.

**Table 3:** Multivariate COX regression analysis of OS.

Characteristics	*p*-value	HR(95%CI)
BMI	0.008	0.94 (0.89- 0.98)
LDL-C	0.631	0.97 (0.88- 1.08)
HDL-C	0.035	0.84 (0.72- 0.99)
Tumor size	0.105	0.93 (0.86- 1.01)
Smoking (Yes vs No)	0.006	1.92 (1.21- 3.07)
Histological grade (Well)	0.747	0.90 (0.49- 1.67)
Histological grade (Well-moderate)	0.288	1.45 (0.73- 2.86)
Histological grade (Moderate-poor)	0.154	0.66 (0.38- 1.17)
Histological grade (Poor)	0.096	1.51 (0.93- 2.46)
Child-Pugh grade (B vs A)	0.173	1.30 (0.89- 1.89)
Lymphatic vessel Invasion (Yes vs No)	0.003	1.89 (1.25- 2.87)
Nerve Invasion (Yes vs No)	0.001	2.00 (1.33- 2.99)
Multiple Tumors (Yes vs No)	0.311	1.26 (0.81- 1.96)
AJCC	0.003	1.46 (1.13- 1.87)

BMI, Body Mass Index; BIL, Bilirubin; AJCC, American Joint Committee on Cancer; LDL-C, Low-Density Lipoprotein Cholesterol; HDL-C, High-Density Lipoprotein Cholesterol; OS, overall survival. *p-value<0.05* is statistically significant.

Pearson correlation analysis demonstrated a significant negative correlation between LDL-C and HDL-C (r = −0.35, 95% CI: −0.47 to −0.22, P < 0.001) (**Figure 4C**). Forward stepwise Cox regression showed that LDL-C alone was not significantly associated with OS (HR = 0.999, P = 0.998; C-index = 0.508). After HDL-C was added to the model, the regression coefficient of LDL-C changed from −0.0001 to −0.038, whereas the C-index increased only slightly from 0.508 to 0.537 (**Supplementary Table 2**). The variance inflation factor (VIF) for LDL-C was 5.21 (**Supplementary Table 3**), and LDL-C remained non-significant after adjustment for HDL-C (P = 0.47).

### Nomogram construction and validation

**Figure 5:**
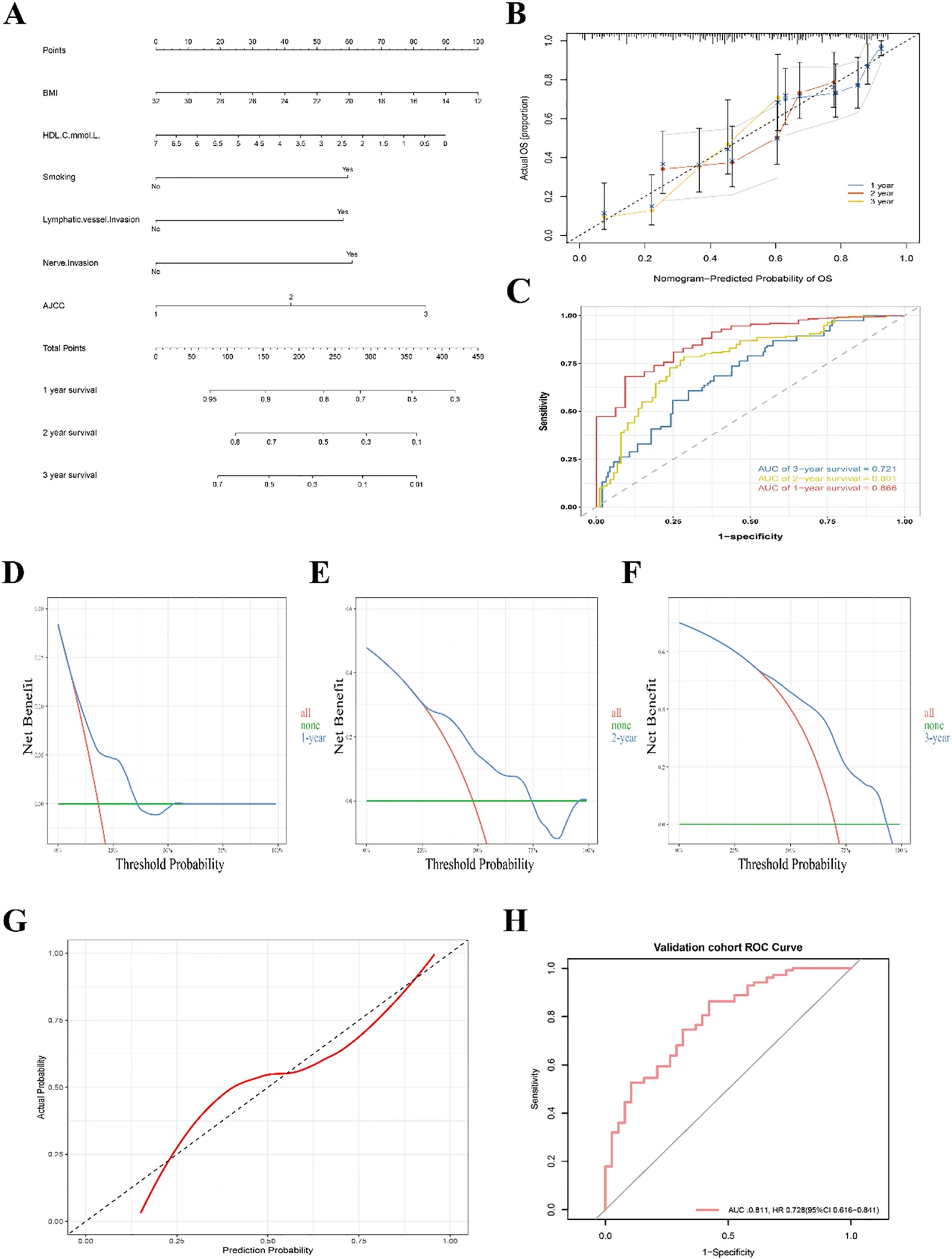
The prognosis prediction model and relevant validation. **(A)** The nomogram of OS. **(B)** The calibration of different years for the model. **(C)** the time-ROC of the model. **(D)** The DCA of 1-year. **(E)** The DCA of 2-year. **(F)** The DCA of 3-year. **(G)** The calibration of the model by validation cohort. **(H)** The ROC of the model by validation cohort. ROC, Receiver operating characteristic; DCA, Decision curve analysis; HDL-C, High-Density Lipoprotein Cholesterol.

Based on the multivariate Cox regression analysis, independent prognostic factors for OS were incorporated into the final prognostic model (**Figure 5A**). Calibration curves demonstrated good agreement between the predicted and observed survival probabilities at 1, 2, and 3 years (**Figure 5B**). The model achieved a C-index of 0.701 (**Supplementary Table 4**), indicating satisfactory predictive performance. The discriminative ability of the model was further evaluated using time-dependent ROC analysis. The AUC values for predicting 1-, 2-, and 3-year OS were 0.866, 0.801, and 0.721, respectively (**Figure 5C**). Decision curve analysis (DCA) demonstrated favorable net clinical benefit across all evaluated time points (**Figures 5D–F**). In addition, the Hosmer–Lemeshow goodness-of-fit test showed good model calibration (P = 0.23; **Supplementary Table 5**).

The contribution of HDL-C to the final model was further assessed using the Akaike information criterion (AIC). Compared with both the empty model (AIC = 348.2) and the model without HDL-C (AIC = 321.5), the final model incorporating HDL-C achieved the lowest AIC (295.2), indicating an improved overall model fit (**Supplementary Table 6**).

The model was subsequently validated in the validation cohort. Calibration analysis demonstrated good agreement between the predicted and observed survival probabilities (**Figure 5G**). Time-dependent ROC analysis yielded an AUC of 0.811, confirming satisfactory discriminative performance in the internal dataset (**Figure 5H**).

## Discussion

The AJCC staging system remains an important tool for prognostic evaluation in cancer patients which was also confirmed in the our study (3). However, with the continuous advancement of prognostic research in ICC, accumulating evidence has suggested that relying solely on anatomical features has limitations in predicting overall survival, particularly in patients who undergo curative resection (16).

Through Cox regression modeling, our study identified low HDL-C as an independent adverse prognostic factor in ICC patients following surgery. Consistent with our findings, Shu et al. reported that preoperative serum HDL-C levels were significantly associated with long-term survival in patients with ICC. Specifically, patients with reduced HDL-C levels exhibited a higher incidence of significantly shorter overall survival compared with those with higher HDL-C levels. Multivariate analysis further confirmed low HDL-C as an independent predictor of poor prognosis, suggesting that HDL-C may serve as a simple and reliable biomarker for postoperative risk stratification in ICC. The prognostic value of HDL-C has also been demonstrated in other malignancies. Lin et al. found that patients with cervical cancer exhibited significantly elevated serum LDL-C levels and reduced HDL-C levels compared with healthy controls, and that the combination of high LDL-C and low HDL-C was associated with advanced clinicopathological features and unfavorable survival outcomes. Similarly, a recent study investigating plasma lipid profiles in patients with head and neck squamous cell carcinoma (HNSCC) reported that decreased HDL-C levels were significantly associated with poorer OS. After adjustment for conventional clinicopathological variables, HDL-C remained an independent prognostic factor, highlighting the clinical relevance of systemic lipid metabolism in tumor progression. Collectively, these findings suggest that dysregulated lipid metabolism, particularly reduced circulating HDL-C, may represent a common metabolic characteristic associated with aggressive tumor behavior across multiple cancer types (14).

Several biological mechanisms may explain the association between low HDL-C levels and adverse oncological outcomes. HDL possesses well-established anti-inflammatory, antioxidant, and immunomodulatory properties. It facilitates reverse cholesterol transport, thereby limiting intracellular cholesterol accumulation, which is essential for membrane synthesis, lipid raft formation, and oncogenic signaling in rapidly proliferating tumor cells (17). Reduced HDL-C levels may therefore promote tumor growth by increasing cholesterol availability and enhancing proliferative signaling pathways. In addition, HDL inhibits oxidative stress and chronic inflammation by neutralizing oxidized lipids and suppressing pro-inflammatory cytokine production. Given that persistent inflammation is a recognized driver of ICC progression, decreased HDL-C may contribute to a tumor-promoting microenvironment (18, 19).

These findings are generally consistent with our results and suggest a potential association between increased cancer risk, unfavorable cancer prognosis, elevated LDL-C levels, and reduced HDL-C levels (15, 20).

In our study, we can find that the optimal HDL-C cut-off of 2.31 mmol/L is far above the normal adult reference range. Combined with clinical practice, our team infer that due to the liver serves as the central hub for lipid metabolism, malignant transformation of hepatocytes may disrupt both the synthesis and efflux of lipids, potentially accounting for the markedly elevated preoperative HDL-C levels observed in this cohort (21). Otherwise, the team from beijing chaoyang hospital demonstrated that HDL-C ≤ 0.415 mmol/L is an independent risk factor for postoperative prognosis in dCCA patients. The study included 203 individuals and confirmed the optimal cutoff by ROC (22). Compared with our study, it’s obvious that sample size is critical role of cutoff for HDL-C. Our cutoff represents a statistically derived optimum tailored to our specific dataset. Its applicability to other populations or its role as a clinically actionable threshold should be evaluated through external validation in larger, multicenter cohorts. Accordingly, we have explicitly characterized it as the ‘optimal prognostic stratification cutoff within the present study, ‘ rather than endorsing it as a new clinical reference standard (14, 23).

Although LDL-C demonstrated excellent discriminatory ability in ROC analysis, it did not retain independent prognostic significance after multivariable adjustment. To further investigate this discrepancy, we performed additional statistical analyses. Forward stepwise Cox regression showed that LDL-C alone was not independently associated with overall survival, and the regression coefficient of LDL-C changed markedly after HDL-C was introduced into the model, accompanied by a modest increase in model discrimination. In addition, the VIF of LDL-C (5.21) suggested a moderate degree of collinearity between LDL-C and HDL-C. Collectively, these findings indicate that the apparent prognostic value of LDL-C may be influenced by its correlation with HDL-C rather than representing an independent prognostic effect. Therefore, HDL-C was selected for inclusion in the final prognostic model.

By integrating independent prognostic factors into a nomogram, the present study successfully established a visualized tool for predicting the 3-year survival risk of ICC patients following surgery. In the training cohort, the model demonstrated favorable goodness-of-fit (Hosmer–Lemeshow test, p=0.23), predictive accuracy (calibration curves closely approximated the ideal reference line), discriminative ability (all time-dependent ROC AUC values >0.7), and considerable clinical net benefit (DCA showed superior performance over the null model across multiple time points). During internal validation, the model similarly exhibited good stability and generalizability. The calibration curve in the validation cohort closely overlapped with the ideal reference line, while the AUC reached 0.811, indicating that the model maintained satisfactory discriminative performance in an independent dataset. Compared with other model, HDL-C is routinely measured during preoperative biochemical testing, making the model highly accessible and easy to implement in routine clinical practice. This simplicity facilitates implementation in routine clinical practice, particularly in resource-limited settings. Our model integrates a metabolic biomarker with the conventional AJCC staging system, thereby complementing anatomical staging with metabolic information. Nevertheless, unlike the multicenter PIIN model Zhu et al. constructed, our study was based on a single-center retrospective cohort with internal validation only, which may limit its generalizability (6).

Nevertheless, several limitations of the present study should be acknowledged. First, this was a single-center retrospective study with a relatively limited sample size, and we didn’t include patients who underwent adjuvant therapy; therefore, large-scale multicenter studies witch contain adjuvant therapy are still required to further validate and strengthen the present findings. Second, the lack of available tissue and blood samples limited further investigation into metabolic reprogramming mechanisms and were the reason for higher cutoff of HDL-C, which will constitute an important direction of our future research. Third, the serum lipid–based nomogram developed in this study still requires extensive external validation across different populations before it can be broadly applied in clinical practice. Forth, due to excluding patients with diabetes, steatohepatitis, or lipid-lowering medications makes the cohort unrepresentative of real-world ICC patients with metabolic comorbidities.

## Conclusion

Overall, the present study confirmed the prognostic value of incorporating HDL-C into the AJCC staging system in ICC patients following curative resection. Based on these factors, we further established a nomogram model with favorable internal validation performance, providing an important reference for individualized risk stratification and clinical decision-making in this patient population. In the future, the integration of additional molecular biomarkers and immune-related features may further optimize model performance and enhance its potential application in precision medicine.

## Data Availability

The original contributions presented in the study are included in the article/**Supplementary Material**. Further inquiries can be directed to the corresponding authors.
