# Supplementary material for: Development of an HDL-C combined with AJCC staging system for predicting overall survival after curative resection of intrahepatic cholangiocarcinoma without metabolic comorbidities
Source: Front Oncol. 2026 Aug 7;16:1884360. doi: 10.3389/fonc.2026.1884360 (PMC13494928; doi:10.3389/fonc.2026.1884360)
Supplement: Supplementary file 1 [file Table1.docx]

Table S1 Sensitivity Analysis of ECOG

| Characteristics | *p-value* | HR (95%CI) |
| --- | --- | --- |
| BMI | 0.006 | 0.93 (0.89, 0.98) |
| LDL-C | 0.417 | 0.85 (0.57, 1.26) |
| HDL-C | 0.039 | 0.84 (0.81- 0.87) |
| Tumor size | 0.137 | 0.94 (0.87, 1.02) |
| Smoking (Yes vs No) | 0.007 | 1.93 (1.19, 3.12) |
| Histological grade (Well) | 0.702 | 0.89 (0.48, 1.63) |
| Histological grade (Well-moderate) | 0.423 | 1.32 (0.67, 2.63) |
| Histological grade (Moderate-poor) | 0.24 | 0.71 (0.40, 1.26) |
| Histological grade (Poor) | 0.065 | 1.58 (0.97, 2.58) |
| Child-Pugh grade (B vs A) | 0.178 | 1.29 (0.89, 1.88) |
| Lymphatic vessel Invasion (Yes vs No) | 0.002 | 1.98 (1.28, 3.06) |
| Nerve Invasion (Yes vs No) | 0.002 | 1.93 (1.28, 2.90) |
| Multiple Tumors (Yes vs No) | 0.274 | 1.28 (0.82, 2.00) |
| AJCC | 0.004 | 1.44 (1.12, 1.85) |
| ECOG | 0.501 | 1.20 (0.71, 2.03) |

**Notes:** C-index 0.692; *p-value*<0.05 is statistically significant. **Abbreviations:** BMI, Body Mass Index; BIL, Bilirubin; ECOG, Eastern Cooperative Oncology Group; CEA, Carcinoma Embryonic Antigen; CA199, Carbohydrate Antigen 199; AJCC, American Joint Committee on Cancer.

Table S2 Forward stepwise Cox regression for LDL-C and HDL-C

|  | Model 1 | Model 2 |
| --- | --- | --- |
| Characteristic | LDL-C | LDL-C+HDL-C |
| HR | -0.0001 | -0.038 |
| *P* | 0.998 | 0.4733 |
| C-index | 0.508 | 0.537 |

Table S3 Variance Inflation Factor for LDL-C and HDL-C

|  | LDL-C | HDL-C |
| --- | --- | --- |
| VIF | 5.21 | 5.31 |

Table S4 C-index of Nomogram

| C | Se (C) |
| --- | --- |
| 0.701 | 0.026 |

Table S5 Hosmer and Lemeshow test

| X-squared | df | *p*-value |
| --- | --- | --- |
| 10.518 | 8 | 0.23 |

Table S6 AIC of 3 Models

|  | Empty model | Model without HDL-C | Model with HDL-C |
| --- | --- | --- | --- |
| AIC | 348.2 | 321.5 | 295.2 |
